# miR-148a-3p represses proliferation and EMT by establishing regulatory circuits between ERBB3/AKT2/c-myc and DNMT1 in bladder cancer

**DOI:** 10.1038/cddis.2016.373

**Published:** 2016-12-01

**Authors:** Xiao Wang, Zhen Liang, Xin Xu, Jiangfeng Li, Yi Zhu, Shuai Meng, Shiqi Li, Song Wang, Bo Xie, Alin Ji, Ben Liu, Xiangyi Zheng, Liping Xie

**Affiliations:** 1 Department of Urology, The First Affiliated Hospital, School of Medicine, Zhejiang University, Hangzhou, Zhejiang Province, People's Republic of China; 2 Department of Urology, Tongde Hospital of Zhejiang Province, Hangzhou, Zhejiang Province, People's Republic of China; 3 Department of Urology, Zhejiang Provincial People's Hospital, Hangzhou, Zhejiang Province, People's Republic of China

## Abstract

miR-148a-3p downregulation has emerged as a critical factor in cancer progression yet, the underlying mechanisms of miR-148a-3p expression pattern and its function in bladder cancer remains to be elucidated. Here, we illustrate that miR-148a-3p is frequently downregulated in bladder cancer and that its expression may be regulated by DNA methylation. DNA methyltransferase 1 (DNMT1) and miR-148a-3p function in a positive feedback loop in bladder cancer. miR-148a-3p overexpression functions as a tumor suppressor in bladder cancer cells. miR-148a-3p inhibits bladder cancer cell proliferation and epithelial–mesenchymal transition (EMT) by regulating ERBB3/AKT2/c-myc and ERBB3/AKT2/Snail signaling. ERBB3, DNMT1 and AKT2 are downstream miR-148a-3p target genes. Furthermore, the miR-148a-3p/ERBB3/AKT2/c-myc signaling axis establishes a positive feedback loop in the regulation of bladder cancer. Taken together, our study demonstrates novel regulatory circuits involving miR-148a-3p/ERBB3/AKT2/c-myc and DNMT1 that controls bladder cancer progression, which may be useful in the development of more effective therapies against bladder cancer.

Bladder cancer is one of the most common cancers in the world,^1, 2^ and approximately one-third of bladder cancer patients develop locally advanced and metastatic disease.^3^ The 5-year survival rate is lower than 62%.^4^ Therefore, it is of great importance to understand the carcinogenic mechanisms and develop novel therapeutic targets of bladder cancer.

MicroRNAs (miRNAs) are small (20–23 nucleotides) non-coding RNAs that comprise a novel class of target gene regulators, and they act by accelerating the degradation and/or blocking the translation of their target mRNAs.^5, 6^ In our previous studies, we identified a series of miRNAs involved in bladder cancer proliferation, migration and invasion, including miR-26a, miR-101, miR-124-3p, miR-320c, miR-409-3p, miR-490-5p, miR-433 and miR-576-3p.^7, 8, 9, 10, 11, 12, 13, 14^ However, the biological function of miRNAs in bladder cancer is still unclear.

The ERBB3 transmembrane receptor is a member of the human epidermal growth factor receptor (EGFR) family. Activated ERBB3 contributes to cell proliferation, migration and survival.^15^ ERBB3 contains multiple binding sites for p85, the regulatory subunit of phosphoinositide 3-kinase (PI3K). p85 induces direct recruitment and activation of the PI3K pathway by ERBB3.^16^ However, the function of activated ERBB3 and its relationship to downstream signaling in bladder cancer has not been well described.

In our study, we investigated the role of miR-148a-3p, a c-myc inhibited miRNA, in the regulation of bladder cancer proliferation and migration. Furthermore, we identified novel regulatory circuits involving miR-148a-3p/ERBB3/AKT2/c-myc and DNA methyltransferase 1 (DNMT1) in the control of bladder cancer progression.

## Results

### miR-148a-3p is downregulated in bladder cancer

**Figure 1:**
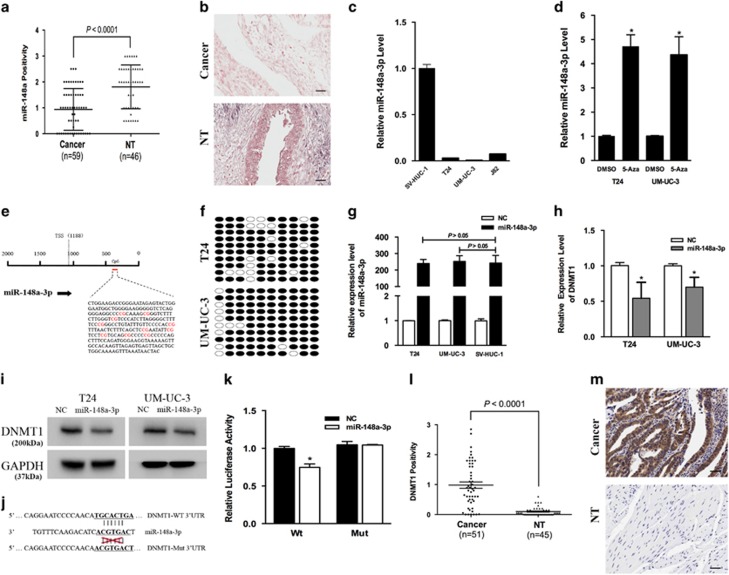
miR-148a-3p is frequently downregulated in bladder cancer and is regulated by DNA methylation. (**a**) Statistical analysis indicated that miR-148a-3p expression was significantly lower in bladder cancer tissues than in adjacent non-tumor tissues. (**b**) Representative images of ISH staining of TMA. miR-148a-3p localized to the cytoplasm. (**c**) miR-148a-3p levels in bladder cancer cell lines (J82, UM-UC-3 and T24) were detected and compared with the non-tumor urothelial cell line SV-HUC-1. (**d**) The demethylating agent 5-Aza-dC stimulated miR-148a-3p expression compared with DMSO-treated samples. (**e**) The regions analyzed by bisulfite-sequencing PCR (BSP) are indicated. (**f**) Methylation profile in T24 and UM-UC-3 cells. The open and filled circles represent the unmethylated and methylated CpG islands, respectively. Ten clones from each cell line were analyzed. (**g**) The fold change of miR-148a-3p level was similar in both bladder cancer cell lines and normal epithelial bladder cell. (**h** and **i**) Decreased DNMT1 expression was observed in miR-148a-3p-transfected T24 and UM-UC-3 cells via qRT-PCR and western blot. (**j**) The miR-148a-3p-targeting sites in the DNMT1 3′-UTR were mutated. (**k**) miR-148a-3p significantly suppressed the luciferase activity of vector that carried the DNMT1 3′-UTR but not control vector. (**l**) Statistical analysis indicated that DNMT1 expression in bladder cancer tissues was significantly higher than that in adjacent non-tumor tissues. (**m**) Representative images of IHC staining of TMA. DNMT1 localized to the nucleus. Error bars represent the S.E. obtained from three independent experiments; *P<0.05. Scale bar=100 *μ*m

ISH analysis demonstrated that miR-148a-3p expression was significantly lower in bladder cancer tissues compared with adjacent non-tumor tissues (*P*<0.001, Figure 1a) and that miR-148a-3p localized to the cytoplasm (Figure 1b). To further evaluate miR-148a-3p expression in bladder cancer, we performed quantitative real-time PCR (qRT-PCR) in T24, UM-UC-3 and J82 cell lines. miR-148a-3p expression was significantly downregulated in all three different bladder cancer cell lines compared with the SV-HUC-1 cell line (Figure 1c). The above results were consistent with previously published data, indicating that miR-148a-3p had an important role in bladder cancer progression.

Previous studies have demonstrated that DNA methylation can regulate miR-148a-3p expression.^17, 18^ Treatment with the DNA methyltransferase inhibitor 5-Aza-2′-deoxycytidine (5-Aza) significantly increased miR-148a-3p expression in the T24 and UM-UC-3 cell lines (Figure 1d). We predict the transcription start site (TSS) of miR-148a-3p using the miRStart database (http://mirstart.mbc.nctu.edu.tw/home.php). The results indicated the miR-148a-3p TSS was located 1188-bp upstream of its precursor (Figure 1e). We next used the CpG Island Searcher Program (http://www.urogene.org/methprimer) to identify a CpG island 0–500-bp upstream of the miR-148a-3p TSS. We then conducted sodium bisulfite-sequencing assays to assess the methylation status of the predicted CpG island (Figure 1e). We found that 84.0 and 88.0% of CpG islands were methylated in both bladder cancer cell lines, which was markedly increased. These data further verified the involvement of methylation in miR-148a-3p silencing (Figure 1f).

DNMT1 has a strong preference for hemimethylated over unmethylated DNA, and it maintains DNA methylation. We first overexpressed miR-148a-3p in both bladder cancer cell lines and normal epithelial bladder cell, and found the fold change of miR-148a-3p level was similar in these cell lines (Figure 1g). After treatment with miR-148a-3p, DNMT1 mRNA and protein expression levels decreased (Figures 1h and i). We also identified DNMT1 as a downstream target of miR-148a-3p using the TargetScan (http://www.targetscan.org) online database. We next synthesized the 3′-untranslated region (3′-UTR) of DNMT1 and cloned it downstream of the pmirGLO dual-luciferase miRNA target expression vector. We also generated a vector with mutated DNMT1-binding sites (Figure 1j). 293T cells transfected with the WT-3′-UTR vector and miR-148a-3p showed significantly decreased luciferase activity (Figure 1k). These results indicate that DNMT1 is a direct target of miR-148a-3p. Immunohistochemistry (IHC) analysis indicated that DNMT1 expression was significantly higher in bladder cancer tissues compared with adjacent non-tumor tissues (*P*<0.001, Figure 1l) and that DNMT1 localized to the nucleus (Figure 1m). Clinical characteristics of the bladder cancer patients were listed in Table 1.

### miR-148a-3p overexpression inhibits cell proliferation

**Figure 2:**
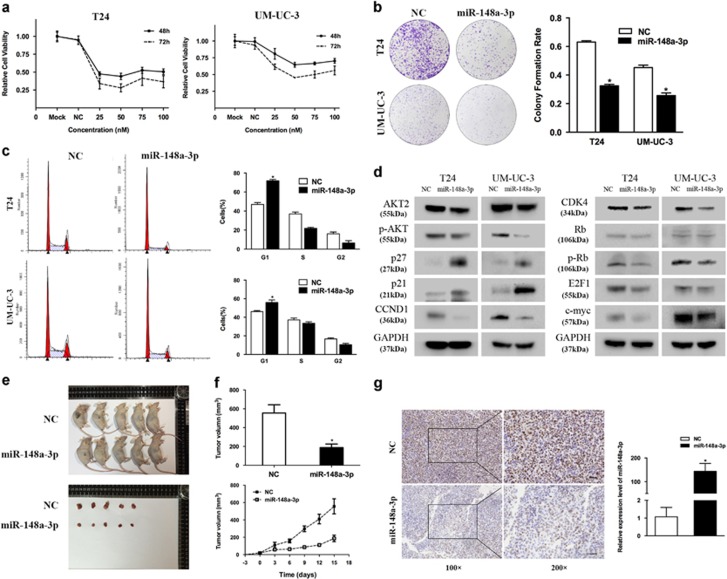
Effect of miR-148a-3p on bladder cancer cell proliferation. (**a**) CCK-8 assay. The relative cell viability of the miR-148a-3p-treated groups of T24 and UM-UC-3 cells was lower than that of NC-treated groups (cell viability of 0 nM was regarded as 1.0). (**b**) Colony formation assay (representative wells are presented). The colony formation rate was lower for miR-148a-3p (50 nM)-transfected groups compared with NC (50 nM)-transfected groups. (**c**) Flow cytometric analysis (representative images are presented) of cell cycle distribution. miR-148a-3p overexpression induced a significant accumulation of cells in the G1-phase and blocked entry into G1-S. (**d**) Western blot analysis. miR-148-3p (50 nM) inhibited the cell cycle and the expression of AKT2 signaling-related proteins in T24 and UM-UC-3 cells. (**e**–**g**) Tumor xenograft model. Tumor volumes and growth curves indicated that tumors in the miR-148a-3p group grew more slowly. Decreased Ki-67 expression and increased miR-148a-3p were also detected in miR-148a-3p-treated tumors. Error bars represent the S.E. obtained from three independent experiments; *P<0.05. Scale bar=100 *μ*m

We transfected T24 and UM-UC-3 cell lines with miR-148a-3p mimics and examined the effects on cellular proliferation. CCK-8 and colony formation assays revealed that miR-148a-3p overexpression significantly decreased the growth rate of both cell lines (Figures 2a and b). We further examined the underlying mechanism for miR-148a-3p-suppressed tumor growth using FACS. We observed a significant increase in the percentage of cells in the G1/G0 phase in cells treated with miR-148a-3p (Figure 2c). Consistent with cell cycle arrest, expression of the cell cycle regulators p21 and p27 significantly increased, whereas the expression of G1/S transition regulators CCND1 and CDK4 significantly decreased in miR-148a-3p-treated cells. The expression of downstream effector proteins of cell cycle signaling p-Rb and E2F1 also significantly decreased. In addition, the expression of AKT2 and AKT phosphorylation-modulated c-myc also significantly decreased (Figure 2d). We examined the growth rates of UM-UC-3 bladder cancer cells in the absence or presence of miR-148a-3p overexpression after s.c. implantation into BALB/c mice. miR-148a-3p overexpression resulted in a marked retardation of tumor growth *in vivo* (Figures 2e and f). IHC staining confirmed that tumors derived from miR-148a-3p-treated cells expressed lower level of Ki-67 than the tumors from the NC-treated group (Figure 2g). We also confirmed that tumors derived from miR-148a-3p-treated cells expressed higher level of miR-148a-3p than the tumors from the NC-treated group (Figure 2g). Taken together, these results showed that miR-148a-3p negatively regulated bladder cancer cell growth.

### miR-148a-3p overexpression inhibits cell motility and EMT

**Figure 3:**
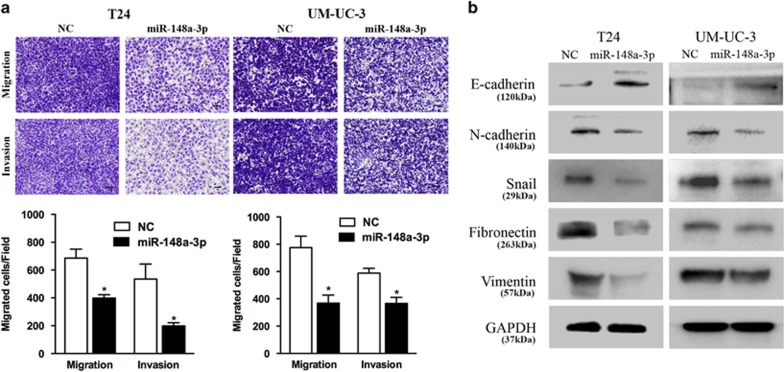
Effect of miR-148a-3p on bladder cancer cell motility. (**a**) Transwell assay (representative micrographs are presented). miR-148a-3p (50 nM) impaired the motility of T24 and UM-UC-3 cells. (**b**) Western blot analysis. miR-148a-3p (50 nM) inhibited EMT-related protein expression in T24 and UM-UC-3 cells. Error bars represent the S.E. obtained from three independent experiments; *P<0.05. Scale bar=100 *μ*m

We found that miR-148a-3p significantly suppressed T24 and UM-UC-3 cell migration and invasion ability in Transwell assays (Figure 3a). Epithelial–mesenchymal transition (EMT) is a key event in cancer invasion and metastasis. We analyzed the effect of miR-148a-3p on EMT by detecting the expression of EMT protein markers. miR-148a-3p overexpression increased the expression of the epithelial marker E-cadherin and decreased the expression of N-cadherin, Fibronectin, Vimentin (mesenchymal markers) and Snail (EMT-related transcription factors). We further explored the underlying mechanism for miR-148a-3p-suppressed EMT (Figure 3b). It has been reported that AKT activation increases Snail nuclear localization and transcriptional activity, thus inducing cell migration and EMT. Upon treatment with miR-148a-3p, phosphorylated AKT expression significantly decreased in bladder cancer cells (Figure 2d). These results indicated that miR-148a-3p could suppress the EMT phenotype of T24 and UM-UC-3 cells by regulating AKT2/Snail signaling.

### ERBB3 and AKT2 are direct miR-148a-3p targets

**Figure 4:**
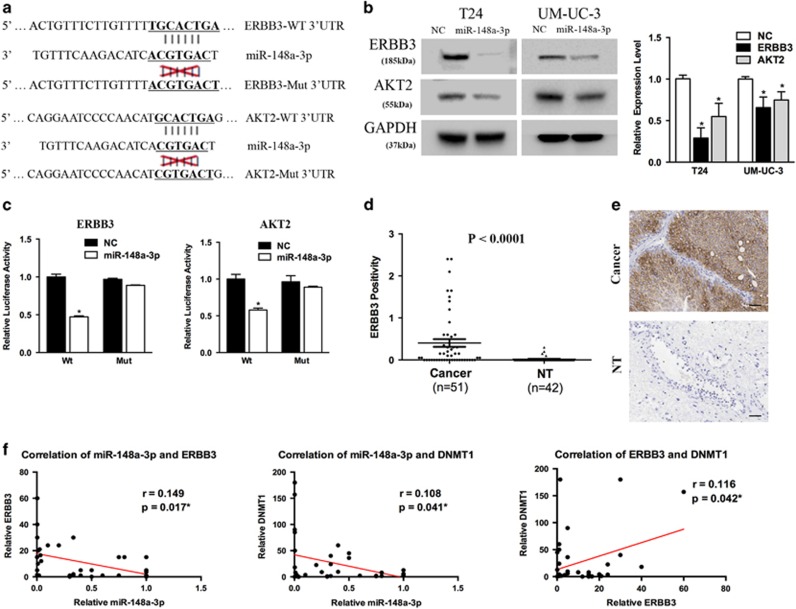
ERBB3 and AKT2 are direct miR-148a-3p targets. (**a**) The miR-148a-3p- targeting sites in ERBB3 and AKT2 3′-UTRs were mutated. (**b**) Decreased ERBB3 and AKT2 expression was observed in miR-148a-3p-transfected T24 and UM-UC-3 cells via qRT-PCR and western blot. (**c**) miR-148a-3p significantly suppressed the luciferase activity of vector that carried the ERBB3 and AKT2 3′-UTRs but not control vector. (**d**) Statistical analysis indicated that ERBB3 expression was significantly higher in bladder cancer tissues than in adjacent non-tumor tissues. (**e**) Representative images of IHC staining of TMA. ERBB3 localized to the membrane. (**f**) miR-148a-3p was significantly negatively correlated with ERBB3 (*r*=0.149, *P*=0.017) and DNMT1 (*r*=0.108, *P*=0.041). ERBB3 was significantly negatively correlated with DNMT1 (*r*=0.116, *P*=0.042). Error bars represent the S.E. obtained from three independent experiments; *P<0.05. Scale bar=100 *μ*m

To identify potential downstream targets of miR-148a-3p, we used the TargetScan (http://www.targetscan.org) and miRanda (http://www.microrna.org/microrna/hpme.do) online databases for analysis. Considering the candidate target genes predicted by the two online databases and the function of miR-148a-3p, we chose ERBB3 and AKT2 as candidate targets (Figure 4a). Previous studies have shown that ERBB3 and AKT2 are key cell motility and proliferation regulators, respectively.^19^ We examined decreased expression of both ERBB3 and AKT2 in miR-148a-3p-treated bladder cancer cells at both the mRNA and protein levels (Figure 4b).

We next performed luciferase reporter assays to determine whether miR-148a-3p directly interacted with the 3′-UTRs of ERBB3 and AKT2. miR-148a-3p overexpression significantly decreased the relative luciferase activity of ERBB3 and AKT2, and the luciferase activity of the mutated vectors was unaffected by miR-148a-3p overexpression (Figure 4c). Collectively, these data support the notion that ERBB3 and AKT2 are direct downstream targets of miR-148a-3p.

IHC analysis indicated that ERBB3 expression was significantly higher in bladder cancer tissues than in adjacent non-tumor tissues (*P*<0.001, Figure 4d) and that ERBB3 localized to the membrane (Figure 4e). We also analyzed the AKT2 expression pattern in the same samples. However, AKT2 expression pattern in bladder cancer tissues was similar to that in adjacent non-tumor tissues (*P*>0.05). We further searched omcomine online database (www.oncomine.org), and found AKT2 expression patterns were not consistent in different studies. Therefore, we need more clinical samples to further verify AKT2 expression pattern in the future.

We conducted *in vivo* experiments to examine the expression pattern of ERBB3, DNMT1, AKT2, c-myc and N-cadherin. IHC staining confirmed that tumors derived from the miR-148a-3p-treated cells expressed lower levels of ERBB3, DNMT1, AKT2, c-myc and N-cadherin compared with tumors from the NC-treated group (Supplementary Figure S1).

We further compared the expression of miR-148a-3p, DNMT1 and ERBB3 in bladder cancer tissues. As shown in Figure 4f, miR-148a-3p was significantly negatively correlated with ERBB3 (*r*=0.149, *P*=0.017) and DNMT1 (*r*=0.108, *P*=0.041) expression. Moreover, we observed significant positive correlation between ERBB3 and DNMT1 (*r*=0.116, *P*=0.042).

Kaplan–Meier survival curves indicated that the protein ERBB3 expression was significantly associated with a poor overall survival rate of bladder cancer patients (*P*=0.026, Supplementary Figure S2). Finally, in a multivariate Cox model including age, gender, tumor grade, lymph node invasion, ERBB3 expression, DNMT1 expression and miR-148a-3p expression, we found that ERBB3 overexpression was a poor independent prognostic factor for the overall survival in patients with bladder cancer (*P*=0.044, Table 2).

### ERBB3 repression inhibits cell proliferation and EMT

**Figure 5:**
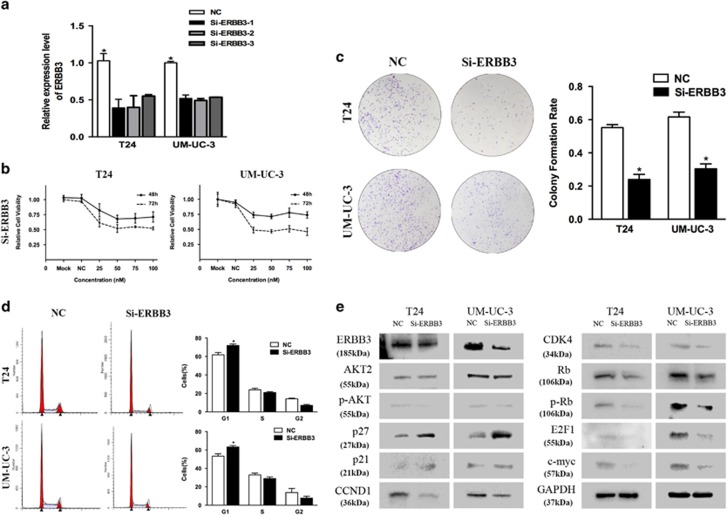
ERBB3 knockdown suppresses bladder cancer cell proliferation. (**a**) Three different and effective siRNAs were used in our studies to avoid off-target phenomena. (**b**) CCK-8 assay. The relative cell viability of the Si-ERBB3 of T24 and UM-UC-3 cells was lower than that of NC-treated groups (cell viability of 0 nM was regarded as 1.0). (**c**) Colony formation assay (representative wells are presented). The colony formation rate was lower for Si-ERBB3 (50 nM)-transfected groups compared with NC (50 nM)-transfected groups. (**d**) Flow cytometric analysis (representative images are presented) of cell cycle distribution. ERBB3 knockdown induced a significant accumulation of cells in the G1-phase and blocked entry into G1-S. (**e**) Western blot analysis. Si-ERBB3 (50 nM) inhibited the cell cycle and AKT2 signaling-related proteins in T24 and UM-UC-3 cells. Error bars represent the S.E. obtained from three independent experiments; *P<0.05

**Figure 6:**
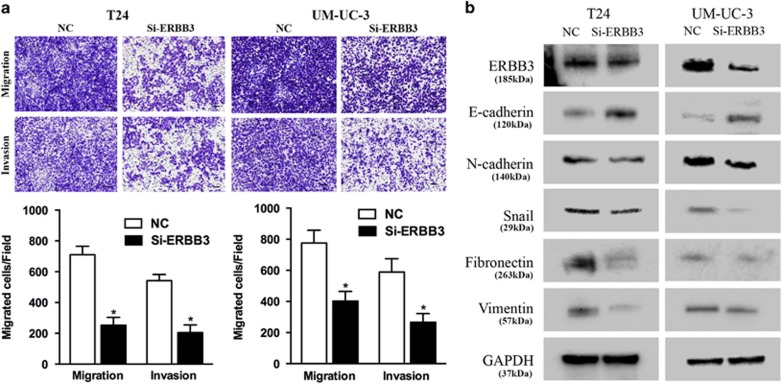
ERBB3 knockdown suppresses bladder cancer cell motility. (**a**) Transwell assay (representative micrographs are presented). Si-ERBB3 (50 nM) impaired T24 and UM-UC-3 cells motility. (**b**) Western blot analysis. Si-ERBB3 (50 nM) inhibited EMT-related protein expression in T24 and UM-UC-3 cells. Error bars represent the S.E. obtained from three independent experiments; *P<0.05. Scale bar=100 *μ*m

The precise role of ERBB3 in bladder cancer is unclear. To avoid off-target phenomena, we use three different and effective siRNAs in our studies (Figure 5a). The following results were obtained from one of the three siRNAs as a representative. We first analyzed the effect of ERBB3 on cell proliferation. CCK-8 assays showed that ERBB3 knockdown by Si-ERBB3 inhibited T24 and UM-UC-3 cell growth at different concentrations and time points (Figure 5b). In parallel, the colony-forming ability of bladder cancer cells decreased, as the colony formation capacity of Si-ERBB3-transfected cells was much worse than that of cells transfected with NC (Figure 5c). In addition, we observed an increase in the percentage of cells in the G1/G0 phase in Si-ERBB3-transfected cells compared with NC-transfected cells (Figure 5d). Consistent with the cell cycle arrest phenomenon, the expression of p21 and p27 significantly increased, whereas CCND1 and CDK4 protein expression significantly decreased in Si-ERBB3-treated cells. p-Rb, E2F1, AKT2 and c-myc expression significantly decreased in Si-ERBB3-treated cells (Figure 5e). In addition, ERBB3 expression was also included as a control showing the silenced target. We performed Transwell assays to verify the role of ERBB3 in cell motility. We found that Si-ERBB3 significantly suppressed T24 and UM-UC-3 cell migration and invasion ability (Figure 6a). These data suggest that ERBB3 can regulate bladder cancer cell proliferation and motility.

As expected, ERBB3 downregulation also inhibited the AKT2/Snail pathway. Si-ERBB3-transfected cells exhibited decreased expression of phosphorylated AKT, N-cadherin, Fibronectin, Vimentin and Snail, as well as increased expression of E-cadherin (Figures 5e and 6b). In addition, ERBB3 expression was also included as a control showing the silenced target. Collectively, these data suggest that repression of ERBB3 can suppress the EMT phenotype of T24 and UM-UC-3 cells, at least in part, by regulating AKT2/Snail signaling.

### AKT2 repression inhibits cell proliferation

The precise role of AKT2 in bladder cancer is limited. To avoid off-target phenomena, we use three different and effective siRNAs in our studies (Supplementary Figure S3A). The following results were obtained from one of the three siRNAs as a representative. We first analyzed the effect of AKT2 on cell proliferation. CCK-8 assays showed that AKT2 knockdown by Si-AKT2 inhibited T24 and UM-UC-3 cell growth at different concentrations and time points (Supplementary Figure S3B). In parallel, the colony-forming ability of bladder cancer cells decreased, as the colony formation capacity of Si-AKT2-transfected cells was much worse than that of cells transfected with NC (Supplementary Figure S3C). In addition, we observed an increase in the percentage of cells in the G1/G0 phase in Si-AKT2-transfected cells compared with NC-transfected cells (Supplementary Figure S3D). Consistent with the cell cycle arrest phenomenon, AKT2, p-AKT and c-myc expression significantly decreased in Si-AKT2-treated cells (Supplementary Figure S3E).

### Inhibition of miR-148a-3p expression partially rescues the Si-ERBB3-induced suppression of cell proliferation and EMT

**Figure 7:**
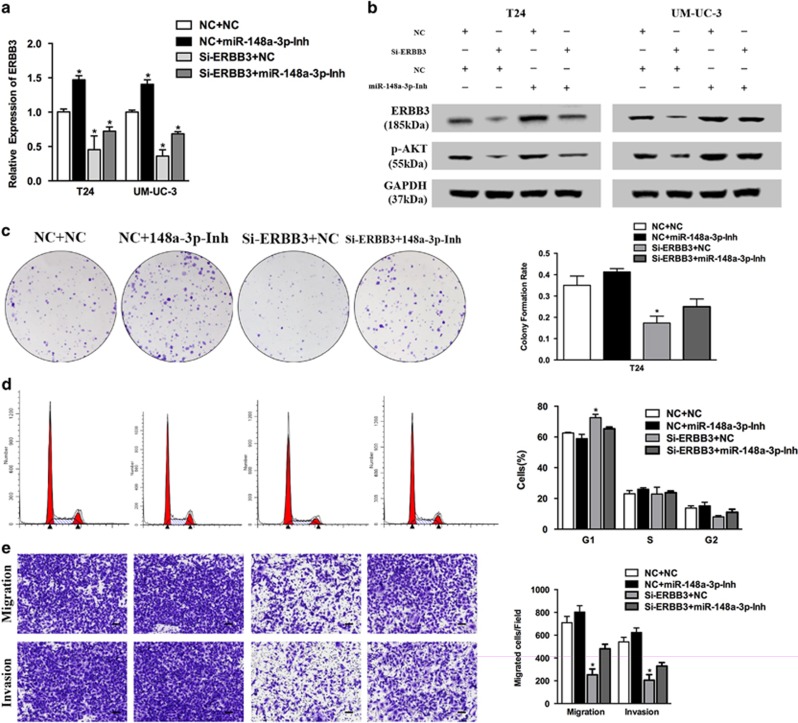
Inhibition of miR-148a-3p expression partially rescues Si-ERBB3-induced suppression of cell proliferation and EMT. (**a** and **b**) Co-transfection of miR-148a-3p-Inh attenuated the ERBB3 expression inhibited by Si-ERBB3 at the mRNA and protein level in both bladder cancer cell lines. Similar p-AKT protein expression pattern was also observed. (**c**–**e**) Transfection of miR-148a-3p-Inh partially but significantly rescued the Si-ERBB3-induced inhibition of cell proliferation, cell cycle and motility in T24 cell line. Error bars represent the S.E. obtained from three independent experiments; *P<0.05. Scale bar=100 *μ*m

We performed rescue experiments to test whether miR-148a-3p inhibition could abrogate the inhibition of cell proliferation and motility by Si-ERBB3. In parallel, we co-transfected miR-148a-3p-Inh to attenuate the ERBB3 mRNA and protein expression inhibited by Si-ERBB3 in both bladder cancer cell lines (Figures 7a and b). The same p-AKT protein expression pattern was also observed (Figure 7b). miR-148a-3p-Inh transfection partially, but significantly, promoted cell proliferation and motility inhibited by Si-ERBB3 (Figures 7c and e and Supplementary Figures S4A-S4C). Thus, we confirmed that ERBB3 is a key mediator of miR-148a-3p tumor suppression function in bladder cancer.

### miR-148a-3p/ERBB3/AKT2/c-myc establishes a positive feedback loop to regulate bladder cancer

**Figure 8:**
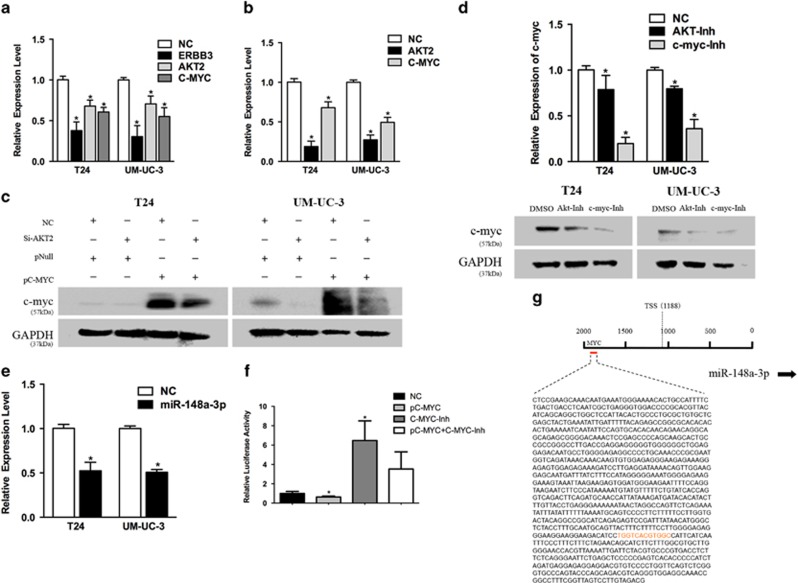
miR-148a-3p/ERBB3/AKT2/c-myc established a regulatory circuit in bladder cancer regulation. (**a**) Transfection of Si-ERBB3 decreased the expression level of its downstream gene AKT2 and c-myc by qRT-PCR. (**b**) Transfection of Si-AKT2 decreased the phosphorylation level of AKT and expression of c-myc. (**c**) Overexpression of c-myc was able to, at least partially, rescue the level of c-myc in the presence of Si-AKT2. (**d**) Both AKT-Inh and c-myc-Inh decreased the expression of c-myc on both mRNA and protein levels. (**e**) qRT-PCR. c-myc overexpression decreased miR-148a-3p expression. (**f**) c-myc overexpression in HEK293T cells increased the luciferase activity of the miR-148a-3p promoter. Co-treatment with c-myc-Inh diminished the increase in luciferase activity. (**g**) c-myc-binding region in the miR-148a-3p promoter region. Error bars represent the S.E. obtained from three independent experiments; *P<0.05

Previous studies have shown that ERBB3 regulates AKT phosphorylation to further phosphorylate and activate c-myc by binding to its ligand.^19^ We found that transfection of Si-ERBB3 decreased AKT2 and c-myc expression by qRT-PCR (Figure 8a). Furthermore, transfection of Si-AKT2 also decreased AKT2 and c-myc expression (Figure 8b). We next showed that c-myc overexpression partially rescued c-myc levels in the presence of Si-AKT2 (Figure 8c). Finally, we used negative control (NC), AKT-Inh and c-myc-Inh (positive control) to further verify the relation between AKT2 and c-myc. We found that both AKT-Inh and c-myc-Inh decreased c-myc mRNA and protein expression (Figure 8d).

Unexpectedly, miR-148a-3p expression decreased upon c-myc overexpression (Figure 8e). To further investigate the mechanisms of c-myc in the regulation of miR-148a-3p, we used the JASPAR database (http://jaspar.genereg.net) to predict the c-myc-binding site in the miR-148a-3p promoter region. We identified a c-myc-binding site in the region 600-bp upstream of the miR-148a-3p TSS (TGGTCACGTGGC) (Figure 8g). Therefore, we cloned the miR-148a-3p promoter region (−1 kb) harboring the predicted c-myc-binding sequences into the pGL3 vector. Treatment with pC-MYC decreased miR-148a-3p promoter luciferase activity. Co-treatment with c-myc-Inh diminished the decrease in luciferase activity (Figure 8f).

Collectively, these data suggest that miR-148a-3p/ERBB3/AKT2/c-myc establishes a positive feedback loop in bladder cancer regulation, indicating that miR-148a-3p is a promising therapeutic target.

**Figure 9:**
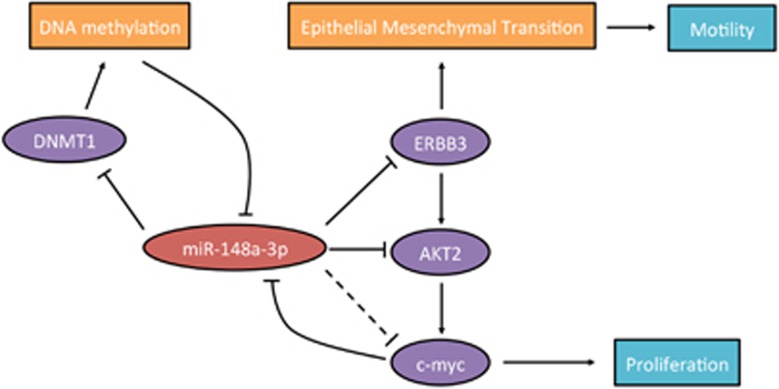
Schematic diagram showing that miR-148a-3p/ERBB3/AKT2/c-myc and DNMT1 established a bi-loop to regulate bladder cancer

A genetic regulatory network is depicted in Figure 9 and summarizes the key findings of our study.

## Discussion

This study investigated the role of miR-148a-3p, a miRNA inhibited by c-myc, in regulating bladder cancer proliferation and migration. We demonstrated novel regulatory circuits involving miR-148a-3p/ERBB3/AKT2/c-myc and DNMT1 that controlled bladder cancer progression. To determine the crucial gene in the above regulatory circuits, we referred to oncomine (https://www.oncomine.org/) and found that ERBB3 expression was increased in infiltrating bladder cancer and superficial bladder cancer compared with adjacent non-cancerous bladder tissues (*P*<0.001). We used bioinformatics and a dual-luciferase reporter system to confirm the relationship between miR-148a-3p and ERBB3. We performed *in vitro* and *in vivo* assays to examine the function of miR-148a-3p/ERBB3 regulatory axis in bladder cancer. Furthermore, we identified ERBB3 as an important prognosis marker in bladder cancer. Therefore, targeting the miR-148a-3p/ERBB3/AKT2/c-myc regulatory circuit may be a novel strategy for the treatment of bladder cancer.

AKT family consists of three members, namely Akt1, Akt2 and Akt3, which share a similar domain structures with one N-terminal pleckstrin homology domain, one central kinase domain and one C-terminal hydrophobic domain.^20^ AKT2 is a significant member of the PI3K/AKT pathway.^21^ Increasing studies demonstrated AKT2 had an important role in cancers as an oncogene.^22^ A previous study demonstrated a mechanistic example of how rewiring signaling pathways after EMT was associated with changes in specific dependencies for proliferation, demonstrating a novel function for ERBB3 in EMT.^19^ High levels of NRG1 stimulated the ERBB2-ERBB3-PI3K-AKT signaling pathway, which was related to the efficacy of ERBB2 inhibitor,^23^ and RTK activation of PI3K-AKT before EMT was important for cancer cell growth and survival.^24^ In this study, we found that ERBB3 repression inhibited EMT in bladder cancer. Furthermore, Si-ERBB3 significantly suppressed T24 and UM-UC-3 cell migration and invasion ability, as demonstrated by Transwell assay. We further verified that ERBB3 repression suppressed the EMT phenotype of T24 and UM-UC-3 cells at least partially by regulating AKT2/Snail signaling, which was consistent with previous experiments.^14^ In addition, ERBB3 repression inhibited bladder cancer proliferation via cell cycle regulation. IHC analysis indicated that ERBB3 expression in bladder cancer tissues was significantly higher compared with that in adjacent non-tumor tissues (*P*<0.001). Elevated ERBB3 expression was significantly associated with a poor overall survival rate of bladder cancer patients, and ERBB3 was an independent prognostic factor for overall survival of bladder cancer patients.

Recent studies have suggested that the dysfunction of miRNAs has a crucial role in carcinogenesis and cancer progression, including bladder cancer.^25, 26^ However, the exact role of miRNA dysfunction in bladder cancer pathogenesis remains controversial. miR-148a-3p is located at the 7p15.2 chromosomal region, which has been implicated to act as a tumor suppressor in many cancers.^27, 28, 29, 30^ Recent studies indicated that miR-148a suppressed the BMP signaling pathway in cancer progression and suppressed cancer cell invasion and metastasis in gastric cancer.^31, 32, 33^ It has been reported that miR-148a suppresses hepatocellular carcinoma cell EMT and metastasis by targeting Met/snail signaling.^34, 35^ In addition, miR-148a-mediated IGF-IR downregulation was accompanied by the attenuation of IGF-IR/IRS1 phosphorylation and AKT/ERK activation.^36^ These reports are consistent with our results identifying a role for miR-148a-3p in bladder cancer. In our research, miR-148a-3p expression was significantly lower in bladder cancer tissues. Furthermore, gain-of-function analyses showed that miR-148a-3p suppressed bladder cancer cell proliferation, migration, and invasion *in vivo* and *in vitro*. These findings have highlighted the cancer suppressor role of miR-148a-3p in bladder cancer. We identified ERBB3 and AKT2 as miR-148a-3p targets, and miR-148a-3p expression was significantly negatively correlated with ERBB3 expression (*r*=0.149, *P*=0.017). In addition, previous studies and our studies indicated that ERBB3 regulated AKT2 phosphorylation to further activate the transcriptional factor c-myc.^19^ Moreover, c-myc downregulated miR-148a-3p. Collectively, these data indicate that miR-148a-3p/ERBB3/AKT2/c-myc establish a positive feedback loop in bladder cancer regulation.

The gene encoding miR-148a-3p is regulated by epigenetic changes, and miR-148a-3p has been reported to interact with DNMT1.^37, 38, 39^ In a previous study, DNMT1 could hypermethylate the miR-148a gene promoters in breast cancer tissues, and DNMT1 overexpression resulted in miR-148a hypermethylation.^27^ In our study, we found that miR-148a-3p surrounding CpG islands were hypermethylated in bladder cancer and that treatment with 5-aza-CdR induced miR-148a-3p overexpression in bladder cancer. We confirmed that DNMT1 was a direct miR-148a-3p target and that miR-148a-3p expression significantly negatively correlated with DNMT1 expression in bladder cancer tissues (*r*=0.108, *P*=0.041). Together, these results indicate that DNMT1 and miR-148a-3p establish a positive feedback loop that is disrupted in both bladder cancer cells and bladder cancer tissues.

Numerous studies have suggested that epigenetic modifications have an important role in the etiology of human diseases.^40, 41^ Abnormal hypermethylation of CpG islands of tumor-suppressor genes and their transcriptional silencing are associated with malignant transformation in cancer.^42^ DNMTs are ubiquitously expressed in normal human tissues and are overexpressed in many types of cancer.^17, 18, 43^ In our study, IHC analysis indicated that DNMT1 expression was significantly higher in bladder cancer tissues than in adjacent non-tumor tissues (*P*<0.001).

In conclusion, we report the following new findings: (1) miR-148a-3p is frequently downregulated in bladder cancer, at least partially due to altered DNA methylation; (2) miR-148a-3p functions as a tumor suppressor in bladder cancer cells; (3) ERBB3, DNMT1 and AKT2 are downstream target genes of miR-148a-3p; (4) miR-148a-3p inhibits bladder cancer cell proliferation and EMT by regulating ERBB3/AKT2/c-myc and ERBB3/AKT2/Snail signaling; (5) miR-148a-3p/ERBB3/AKT2/c-myc form a positive feedback loop in bladder cancer regulation; and (6) DNMT1 and miR-148a-3p establish a positive feedback loop in bladder cancer. Our study identified regulatory loops between miR-148a-3p/ERBB3/AKT2/c-myc and DNMT1 in bladder cancer regulation, which could prove useful in the development of effective and therapies against bladder cancer.

## Materials and Methods

### Cell lines and cell culture

T24, UM-UC-3, J82 and 5637 human bladder cancer cell lines and the nonmalignant bladder epithelial cell line SV-HUC-1 were obtained from the Cell Bank of Type Culture Collection of Chinese Academy of Sciences (Shanghai, China). All cell lines were verified by short tandem repeat (STR) DNA profiling analysis. Cells were cultured as previously reported.^14^

### RNA isolation and real-time PCR

The expression level of miRNAs and genes in the cell lines was calculated by qRT-PCR. RNA was extracted from cell lines with RNAiso plus (TaKaRa, Kusatsu, Japan) and transcribed into cDNA using PrimeScript RT reagent Kit and One Step PrimeScript miRNA cDNA Synthesis Kit (TaKaRa). cDNAs were quantified using SYBR Premix Ex Taq (TaKaRa) with the ABI 7500 fast real-time PCR System (Applied Biosystems, Carlsbad, CA, USA). Small nuclear RNA U6 and GAPDH mRNA were used as internal controls for normalization. All primers sequences are listed in Supplementary Table 1.

### Western blot analysis

Western blot analysis was conducted as previously described.^14^ The following primary immunoblotting antibodies were used: anti-GAPDH, anti-E-cadherin, anti-Fibronectin, anti-Vimentin, anti-Snail, anti-AKT2, anti-p-AKT, anti-Rb, anti-p-Rb (Epitomics, Burlingame, CA, USA), anti-E2F1, anti-CDK4 and anti-DNMT1 (Proteintech, Chicago, IL, USA), anti-N-cadherin, anti-p27, anti-p21, anti-c-myc, anti-ERBB3, and anti-CCND1 (Cell Signaling Technology, Beverly, MA, USA).

### IHC staining

IHC staining was performed as previously described.^14^ Tissue microarrays (TMAs) containing 46 cases with paired tumor and non-tumor tissues and 13 cases without corresponding non-tumor tissues were analyzed (Xinchao Biotech, Shanghai, China). The strength of positivity was semiquantified by considering the intensity and the proportion of positive cells. Tumor tissues from mice were analyzed using the same method.

### *In situ* hybridization (ISH)

A 5′-DIG and 3′-DIG-labeled, locked nucleic acid-incorporated miRNA probe (miRCURY LNA™ Detection probe, Exiqon, Woburn, MA, USA) was used for the visualization of miR-148a in the same bladder cancer TMAs. Paraffin tissue slides were deparaffinized and digested with proteinase K for 6.5 min (15 *μ*g/ml). The slides were then prehybridized in a hybridization solution at 50 °C for 1 h. Tissues were hybridized for 2 days in the presence of 10 ng 3′–5′ DIG-labeled miR-148a LNA probes at 4 °C (500 nM). Slides were washed stringently for 20 min at 50 °C, and an immunological reaction was conducted using anti-DIG-AP Fab fragments according to the manufacturer's protocol. The strength of positivity was semiquantified by considering both the intensity and proportion of positive cells.

### miRNA mimics, miRNA inhibitor and small interfering RNA transfections

miRNA-148a-3p (miR-148a-3p), miRNA-148a-3p inhibitor (miR-148a-3p-Inh), small interfering RNA-ERBB3 (Si-ERBB3), small interfering RNA-AKT2 (Si-AKT2) and NC were purchased from GenePharma (Shanghai, China). Lipofectamine 2000 (Invitrogen, Carlsbad, CA, USA) was used for transfection according to the manufacturer's instructions. All of the small RNAs were used at a final concentration of 50 nM.

### Cell viability assay

Bladder cancer cells (4000 cells per well) were seeded into 96-well plates. After a 24-h incubation, the cells were treated with miR-148a-3p, miR-148a-3p-Inh, Si-ERBB3 or Si-AKT2 for 48- or 72-h incubation. Cell viability assays were performed as previously described.^14^

### Colony formation assay

T24 and UM-UC-3 bladder cancer cells were seeded into a new six-well plate and incubated for 24 h before treatment with 2′-O-methyl-modified miR-148a-3p, Si-AKT2 or Si-ERBB3. Detailed experimental procedures were performed as previously described.^14^

### Cell cycle analysis

Cells were collected 48 h after RNA treatment, and cell cycle analyses were performed with the BD LSRII Flow Cytometer System with FACSDiva Software (BD Bioscience, Franklin Lakes, NJ, USA). The raw data were analyzed via ModFit LT 3.2 software (Verity Software House, Topsham, ME, USA). Detailed experimental procedures were performed as previously described.^26^

### Cell migration and invasion assay

A 24-well Boyden chamber with 8 *μ*m pore size polycarbonate membrane (Corning, NY, USA) was used for evaluating cell migration. Matrigel (BD, Franklin Lakes, NJ, USA) was used to pre-coat the membrane to simulate a matrix barrier for evaluating cell invasion. Transwell assays were performed as previously described.^14^

### Luciferase reporter assays

The putative promoter region of miR-148a-3p (-1 kb) harboring the predicted myc-binding sequences were synthesized by PCR (PrimerSTAR, TaKaRa) and cloned into the pGL3 vector between *Hind*III and *Nhe*I. pENTER-c-myc vector (pC-MYC) or pENTER vector (pNull)-treated T24 or UM-UC-3 bladder cancer cells were co-transfected with the cloned pGL3 vector and renilla luciferase vector in the absence or presence of the c-myc inhibitor (c-myc-Inh, 10058-F4, Selleck, Houston, TX, USA). A total of 48 h after transfection, the relative luciferase activity was calculated using the Dual-Luciferase Reporter Assay System (Promega, Madison, WI, USA).

The 3′-UTRs of DNMT1, ERBB3 and AKT2 containing putative miR-148a-3p target regions were synthesized (Sangon, Shanghai, China) and cloned between the *Sac*I and *Sal*I sites downstream of the luciferase reporter gene in pmirGLO Dual-Luciferase miRNA Target Expression Vector (Promega). In addition, the mutant miR-148a-3p putative target region was generated using the same approach. All insertions were verified by sequencing (Sangon). The dual-Luciferase report assay was performed as previously described.^14^

### 5-Aza treatment of T24 and UM-UC-3 bladder cancer cells

T24 and UM-UC-3 bladder cancer cells were treated with 10 *μ*M 5-Aza (Sigma A3656, St Louis, MO, USA) for 4 days. RNA was extracted and analyzed for miR-148a-3p expression.

### DNA methylation analysis

Genomic DNA from T24 and UM-UC-3 bladder cancer cell lines was bisulfite modified, and the CpG islands were amplified by PCR (primers sequences are listed in Supplementary Table 1). The PCR products were separated by agarose gel electrophoresis (3%), extracted and cloned into the pUC18 T-vector (Sangon). After bacterial amplification of the cloned PCR fragments by standard procedures, 10 clones were sent for DNA sequencing (Sangon).

### Animal experiments

Animal experiments were conducted in accordance with institutional guidelines. Male BALB/c-nude mice (4 weeks old) were purchased from the Shanghai Experimental Animal Center, Chinese Academy of Sciences. Lipofectamine 2000-encapsulated miR-148a-3p or NC was used for injection. Animal experiments were performed as previously described.^26^

### Statistical analysis

All statistics were expressed as the mean±S.E. of three independent experiments. Student's *t*-test or two-way ANOVA was used to evaluate the intergroup difference. Overall survival was analyzed using the Kaplan–Meier method, and the log-rank test was used to estimate the differences between groups. Cox's proportional hazard model was used to assess independent prognostic indicators in multivariate analysis. SPSS for Windows v.16.0 (SPSS, Chicago, IL, USA) and GraphPad Prism 6.0 (GraphPad Software, La Jolla, CA, USA) were used to conduct the relative analyses. *P*<0.05 was considered statistically significant.

## Figures and Tables

**Table 1:** Clinical characteristics of the bladder cancer patients

	**Expression of ERBB3**				
	**Low**		**High**		
	**No. of patients**	**%**	**No. of patients**	**%**	***P*-value**
Total	12		47		
*Age*					
<69	6	50.0	22	46.8	0.843
≥69	6	50.0	25	53.2	
					
*Gender*					
Male	9	75.0	41	87.2	0.547
Female	3	25.0	6	12.8	
					
*Tumor grade*					
Low	0	0	3	6.5	1.000
High	12	100.0	43	93.5	
					
*Tumor stage*					
I–II	6	50.0	22	47.8	0.893
III–IV	6	50.0	24	52.2	
					
*Lymph node invasion*					
Yes	0	0	7	20.6	0.177
No	10	100	27	79.4	
					
*DNMT1*					
High	4	33.3	22	47.8	0.567
Low	8	66.7	24	52.2	
					
*miR-148a-3p*					
High	7	58.3	26	55.3	0.851
Low	5	41.7	21	44.7	

**Table 2:** Univariate and multivariate analyses of predictors of overall survival in bladder cancer patients

**Variables**	**Death**				**Univariable analysis**	**Multivariable analysis**	
	**Yes**	**No**	***N***	**%**	***P*-value**^a^	**HR (95% CI)**	***P*****-value**^b^
*Age*							
<69	13	13	26	44.8	0.963		
≥69	16	16	32	55.2			
							
*Gender*							
Male	23	26	49	84.5	0.298	4.447 (1.350–14.645)	0.014
Female	6	3	9	15.5			
							
*Tumor grade*							
Low	2	1	3	5.3	0.752		
High	27	27	54	94.7			

*Tumor stage*							
I–II	9	19	28	50.0	0.002	6.083 (2.141–17.284)	0.001
III–IV	19	9	28	50.0			

*Lymph node invasion*							
Yes	6	1	7	16.3	0.005		
No	15	21	36	83.7			

*ERBB3*							
High	27	19	46	79.3	0.026	4.575 (1.042–20.084)	0.044
Low	2	10	12	20.7			

*DNMT1*							
High	15	11	26	44.8	0.432		
Low	14	18	32	55.2			

*miR-148-3p*							
High	15	17	32	55.2	0.659		
Low	14	12	26	44.8			

Abbreviations: 95% CI, 95% confidence interval; HR, hazard risk ratio

a Statistical analysis was conducted by Kaplan–Meier method (log-rank test)

b Cox proportional hazards regression
