# Supplementary material for: miR-148a-3p represses proliferation and EMT by establishing regulatory circuits between ERBB3/AKT2/c-myc and DNMT1 in bladder cancer
Source: Cell Death Dis. 2016 Dec 1;7(12):e2503–. doi: 10.1038/cddis.2016.373 (PMC5261009; doi:10.1038/cddis.2016.373)
Supplement: Supplementary Information [file cddis2016373x1.docx]

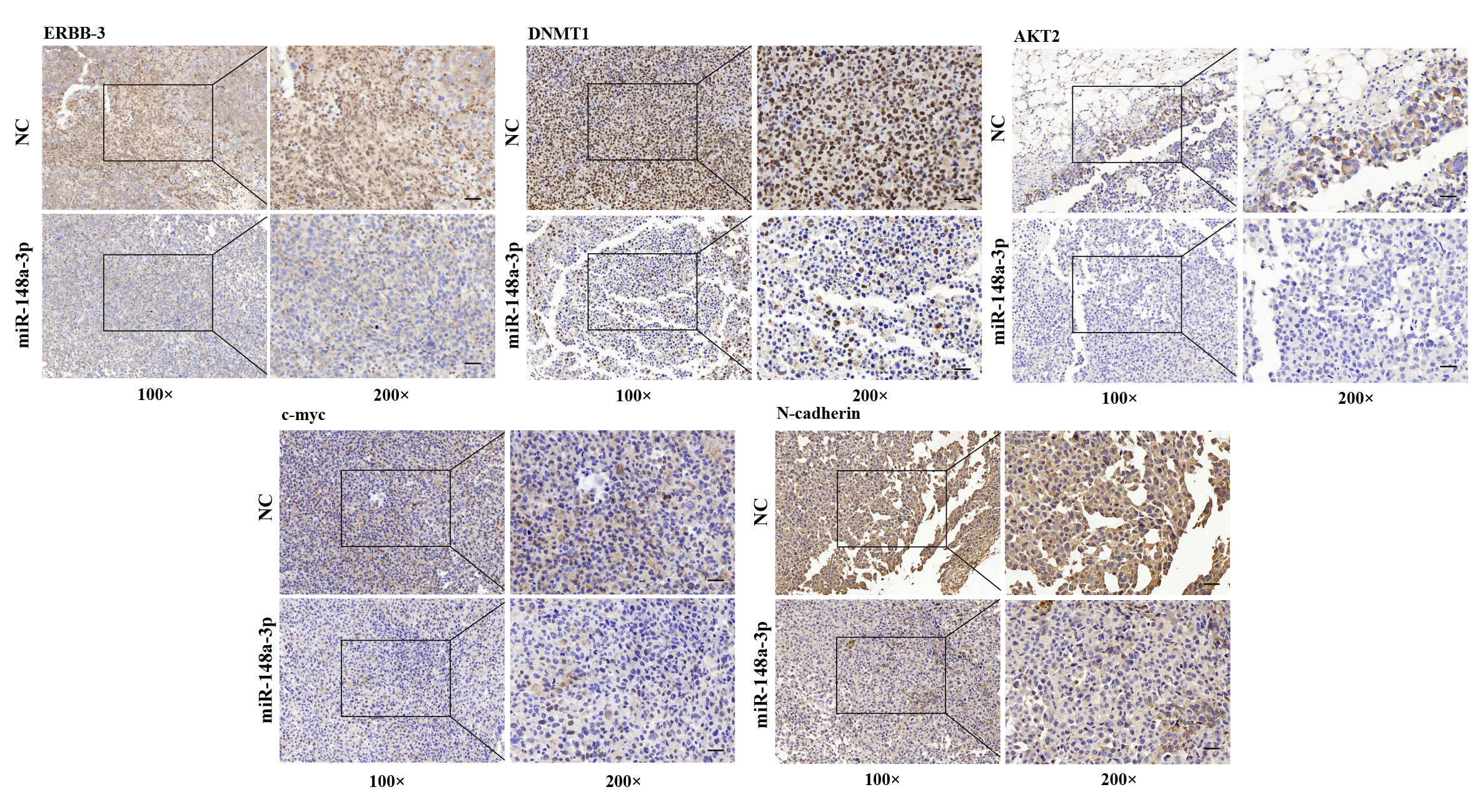


**Figure S1** Tumor xenograft model. Decreased ERBB3, DNMT1, AKT2, c-myc and N-cadherin expression were detected in miR-148a-3p-treated tumors.


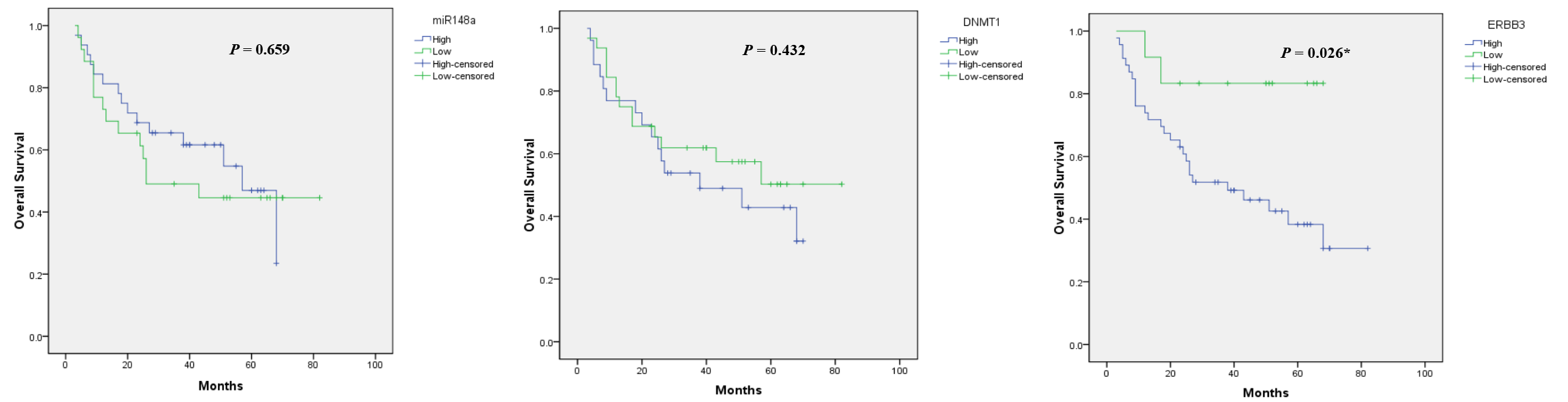


**Figure S2** Kaplan–Meier survival analysis. The protein expression of ERBB3 was significantly associated with the overall survival rate in bladder cancer patients.


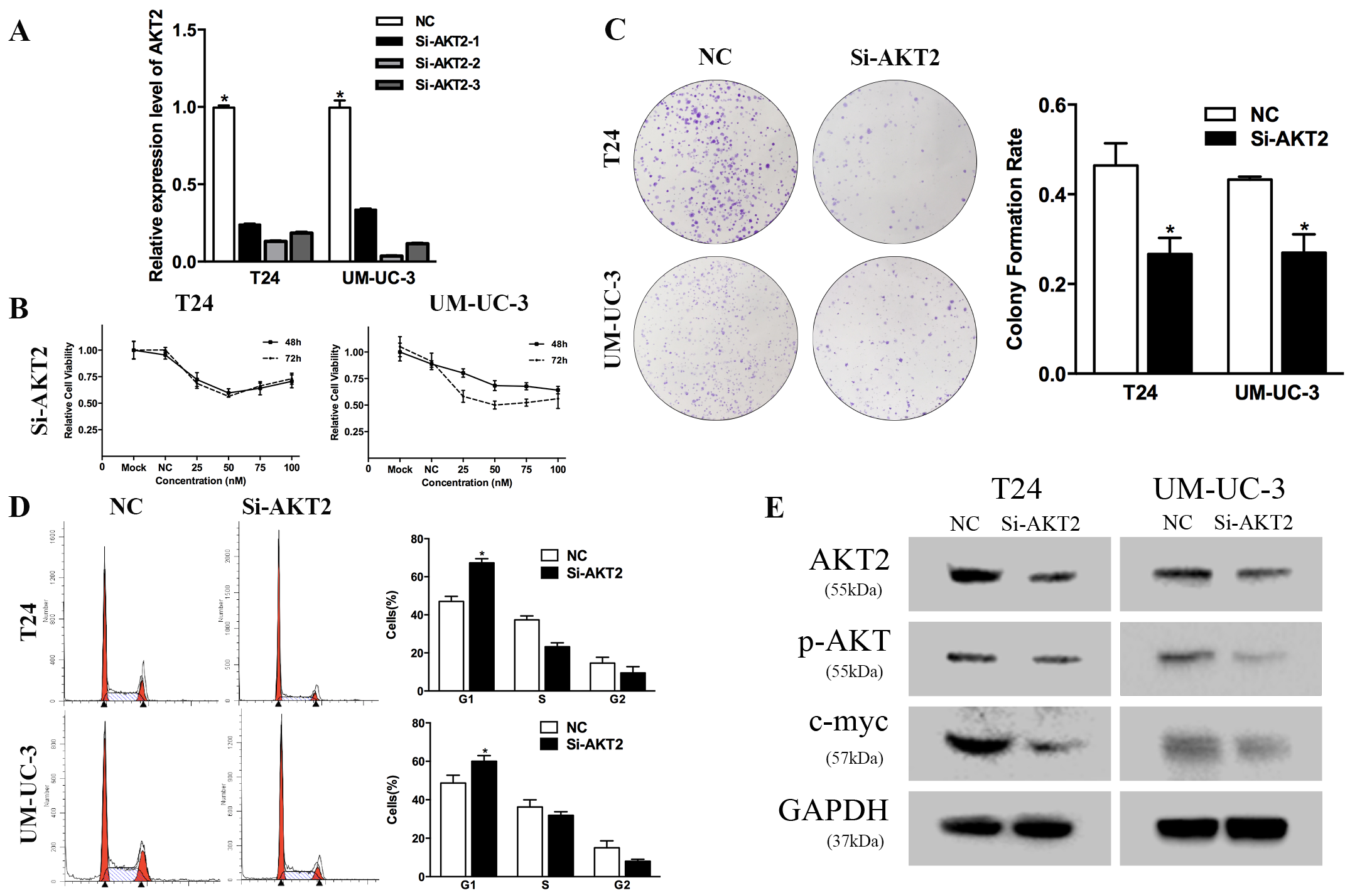
**Figure S3** AKT2 knockdown suppresses bladder cancer cell proliferation. (A) Three different and effective siRNAs were used in our studies to avoid off-target phenomena. (B) CCK-8 assay. The relative cell viability of the Si-AKT2 of T24 and UM-UC-3 cells was lower than that of NC-treated groups (cell viability of 0 nM was regarded as 1.0). (C) Colony-formation assay (representative wells are presented). The colony-formation rate was lower for Si-AKT2 (50 nM)-transfected groups compared to NC (50 nM)-transfected groups. (D) Flow cytometric analysis (representative images are presented) of cell cycle distribution. AKT2 knockdown induced a significant accumulation of cells in the G1-phase and blocked entry into G1-S. (E) Western blot analysis. Si-AKT2 (50 nM) inhibited p-AKT2 and c-myc proteins in T24 and UM-UC-3 cells. Error bars represent the S.E. obtained from three independent experiments; *P<0.05.


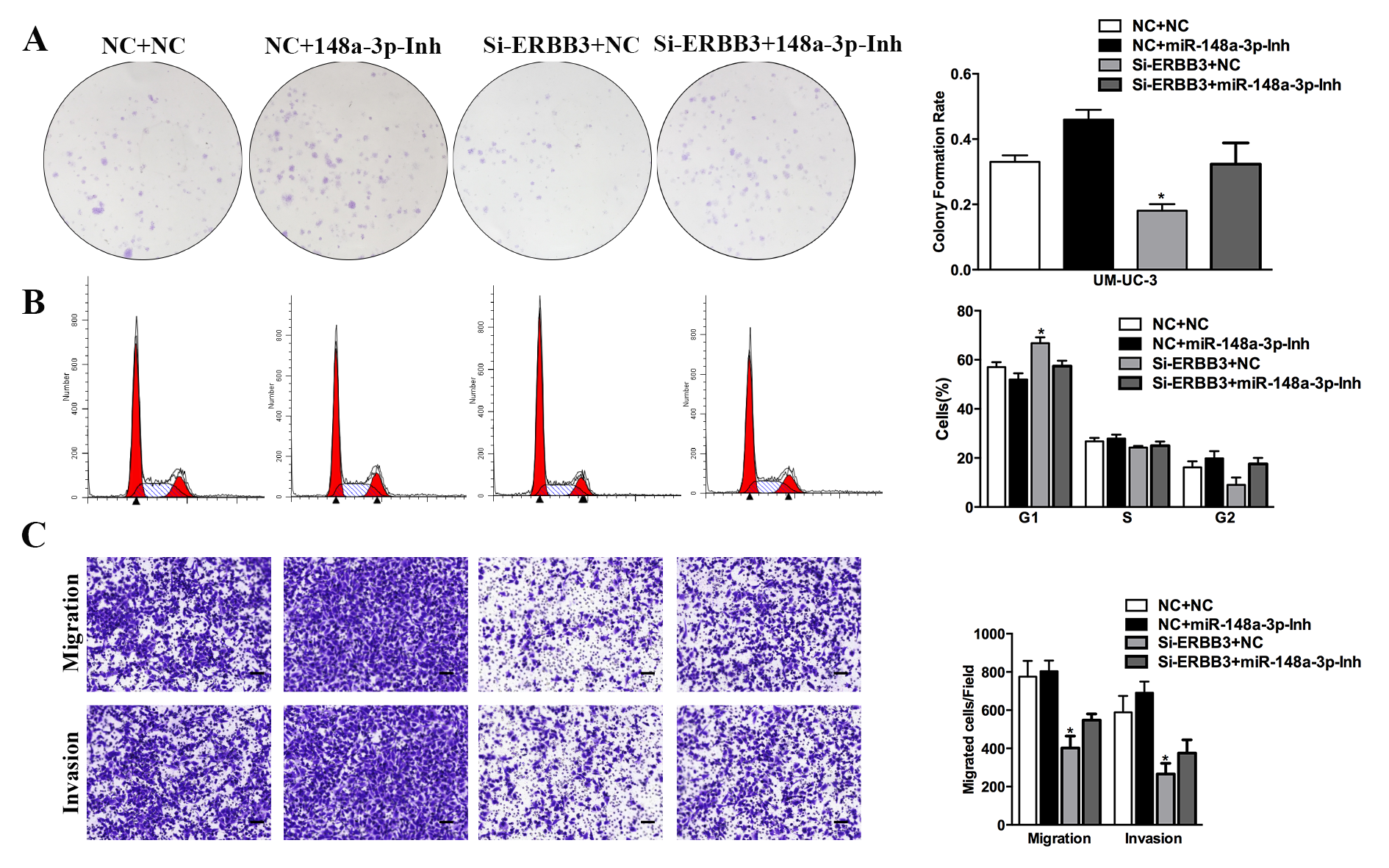


**Figure S4** Inhibition of miR-148a-3p expression partially rescues Si-ERBB3-induced suppression of cell proliferation and EMT in UM-UC-3 cell line. (A, B, C) Transfection of miR-148a-3p-Inh could partially, but significantly, promoted cell proliferation, cell cycle and motility inhibited by Si-ERBB3. Error bars represent the S.E. obtained from three independent experiments; *P<0.05. Scale bar = 100 μm.

Supplementary Table 1 The oligonucleotides used in this study.

| Name^a^ | Sequence (5’->3’) |
| --- | --- |
| miR-148a-3p mimics (sense) | TCAGTGCACTACAGAACTTTGT |
| miR-148a-3p-Inh (sense) | ACAAAGUUCUGUAGUGCACUGA |
| miR-148a probe | ACAAAGTTCTGTAGTGCACTGA |
| NC (sense) | ACUACUGAGUGACAGUAGA |
| Si-AKT2 (sense) | GGGCUAAAGUGACCAUGAATT |
|  | GCUCUUGAGUACUUGCACUTT |
|  | GGUACUUCGAUGAUGAAUUTT |
| Si-ERBB3 (sense) | GUGGAUUCGAGAAGUGACATT |
|  | GCAACAUUGAUGGAUUUGUTT |
|  | CUUGUCCUGUCGAAAUUAUTT |
| Si-DNMT1 (sense) | GCAGGCGGCUCAAAGAUUUTT |
|  | GGAUGAGUCCAUCAAGGAATT |
|  | CCUGCAGAAGAACCUGAAATT |
| miR-148a-3p F | TCAGTGCACTACAGAACTTTGT |
| U6 F | TGCGGGTGCTCGCTTCGGCAGC |
| miR-148a-3p promoter F | tcgaGCTAGCCTCCGAAGCAAACAATGAAA |
| miR-148a-3p promoter R | tcgaAAGCTTCGTCTACAAGGACTAACCGAAA |
| Methylation PCR F | TTGGAAGAT**Y**GGGAATAGAGTATTG |
| Methylation PCR R | TATAACAACTTTTTACCTTCCCATC |
| c-myc F | CCTGGTGCTCCATGAGGAGAC |
| c-myc R | CCTGGTGCTCCATGAGGAGAC |
| ERBB3 F | CTATGAGGCGATACTTGGAACGG |
| ERBB3 R | GCACAGTTCCAAAGACACCCGA |
| AKT2 F | CATCCTCATGGAAGAGATCCGC |
| AKT2 R | GAGGAAGAACCTGTGCTCCATG |
| GAPDH F | AAGGTGAAGGTCGGAGTCA |
| GAPDH R | GGAAGATGGTGATGGGATTT |
| ERBB3-Wt F | cGGGGCACTGTTTCTTGTTTTTGCACTGAATCAAGTCTAACCCCAACAGCg |
| ERBB3-Wt R | tcgacGCTGTTGGGGTTAGACTTGATTCAGTGCAAAAACAAGAAACAGTGCCCCgagct |
| ERBB3-Mut F | cGGGGCACTGTTTCTTGTTTTacgtgactATCAAGTCTAACCCCAACAGCg |
| ERBB3-Mut R | tcgacGCTGTTGGGGTTAGACTTGATagtcacgtAAAACAAGAAACAGTGCCCCgagct |
| AKT2-Wt F | cAGCCTCTGGGGGCTGCCCTC**GTGCACTGA**TGGTTGTGTGGAGTCGGGGGg |
| AKT2-Wt R | tcgacCCCCCGACTCCACACAACCA**TCAGTGCAC**GAGGGCAGCCCCCAGAGGCTgagct |
| AKT2-Mut F | cAGCCTCTGGGGGCTGCCCTCcacgtgactTGGTTGTGTGGAGTCGGGGGg |
| AKT2-Mut R | tcgacCCCCCGACTCCACACAACCAagtcacgtgGAGGGCAGCCCCCAGAGGCTgagct |
| DNMT1-Wt F | cTGGCACCAGGAATCCCCAACATGCACTGATGTTGTGTTTTTAACATGTCg |
| DNMT1-Wt R | tcgacGACATGTTAAAAACACAACATCAGTGCATGTTGGGGATTCCTGGTGCCAgagct |
| DNMT1-Mut F | cTGGCACCAGGAATCCCCAACAacgtgactTGTTGTGTTTTTAACATGTCg |
| DNMT1-Mut R | tcgacGACATGTTAAAAACACAACAagtcacgtTGTTGGGGATTCCTGGTGCCAgagct |

^a^ F, forward primer; R, reverse primer.
